# Prevalence of depressive, anxiety, and stress symptoms and barriers to mental health services among medical students at Jazan University, Saudi Arabia: A cross-sectional study

**DOI:** 10.1097/MD.0000000000041185

**Published:** 2025-01-03

**Authors:** Osama Albasheer, Essam Al Ageeli, Turki I. Aljezani, Khalid A. Bakri, Salman M. Jathmi, Abdullah Maashi, Ahmad Abo Khirat, Ali W. Hakami, Arif A. Haddadi, Suhaila Ali, Amani Abdelmola, Anas Ahmed

**Affiliations:** a Family Medicine, Family and Community Medicine Department, College of Medicine, Jazan University, Jazan, Saudi Arabia; b Medical Genetics, Department of Basic Medical Sciences, Faculty of Medicine, Jazan University, Jazan, Saudi Arabia; c Faculty of Medicine, Jazan University, Jazan, Saudi Arabia; d Community Medicine, Family and Community Medicine Department, College of Medicine, Jazan University, Jazan, Saudi Arabia.

**Keywords:** anxiety, depression, medical students, mental health services, stress

## Abstract

Medical students represent the future of the healthcare workforce. However, the demanding nature of medical education places them at an increased risk of mental health issues. Ensuring their mental well-being is crucial for maintaining a competent and compassionate healthcare system. This study aims to determine the prevalence of depression, anxiety, and stress, along with barriers to accessing mental health services among medical students. A cross-sectional self-administered online survey conducted among medical students of Jazan University, Saudi Arabia, from September 2023 to March 2024. Descriptive and inferential statistical analyses were conducted using International Business Machines Corporation Statistical Package for the Social Sciences version 27.0.1 (Chicago). The study included 390 participants. Median scores for depression, anxiety, and stress were 14.00, 12.00, and 16.00, respectively, with interquartile ranges of 4.00 to 22.00, 4.00 to 20.00, and 6.00 to 24.00. Depression severity categories showed that 38.2% were normal, while 11.5%, 31.8%, 13.6%, and 4.9% experienced mild, moderate, severe, and extremely severe symptoms, respectively. Anxiety severity classifications revealed 37.4% as normal, with 6.2%, 17.4%, 10.0%, and 29.0% falling into mild, moderate, severe, and extremely severe categories, respectively. For stress, 49.2% were normal, while 11.0%, 16.7%, 14.1%, and 9.0% experienced mild, moderate, severe, and extremely severe levels, respectively. Barriers to accessing mental health care included a preference for self-management, confidentiality concerns, societal judgment, and fears of career-related repercussions. This study highlights a high prevalence of depression, anxiety, and stress among medical students at Jazan University, with notable gender differences and symptom severity. Addressing barriers to mental health care, such as confidentiality concerns and societal stigma, is essential to improving service utilization and student well-being.

## 1. Introduction

Medical students face a significantly higher prevalence of mental health problems, including depression, anxiety, burnout, and anorexic tendencies, compared to students in other disciplines.^[1,2]^ The World Health Organization defines a mental health disorder as “a clinically significant disturbance in an individual’s cognition, emotional regulation, or behavior.”^[3]^ Despite the significant mental health challenges medical students encounter, many hesitate to seek assistance due to various barriers. This hesitation, coupled with the demanding nature of their training, can lead to negative outcomes such as substance abuse and an elevated risk of suicide.^[4,5]^

The unique stressors faced by medical students include rigorous academic requirements, exposure to patient suffering and death, a heavy workload, and long study hours.^[4,6]^ These factors contribute to a higher prevalence of mental health issues among medical students compared to their age-matched peers in other fields, with studies reporting a 25% higher rate of depression among medical students.^[4,7,8]^ Moreover, in Saudi Arabia, cultural and familial pressures to excel academically may further increase the risk of depressive symptoms.^[9]^ A study conducted in a Saudi medical college reported that 57% of students experienced stress, highlighting the pervasive nature of mental health challenges in this context.^[10]^ Although several studies have highlighted the high prevalence of mental health problems among medical students globally, few have comprehensively addressed the specific barriers that prevent students from seeking mental health services. A study in Saudi Arabia reported high stress prevalence among medical students, but it did not explore the underlying barriers to accessing mental health care.^[10]^ Similarly, international studies have often focused on prevalence rates without identifying actionable solutions to improve service utilization.^[4,6,8]^ This gap in understanding the specific obstacles faced by medical students – such as stigma, confidentiality concerns, and cultural pressures – limits the development of targeted interventions.

The mission of medical schools is to develop compassionate and competent healthcare professionals. However, the mental health burden faced by students can undermine their ability to fulfill this role and may contribute to higher dropout rates, affecting the healthcare workforce.^[4]^ This study aims to evaluate the prevalence of depression, anxiety, and stress symptoms and identify the barriers preventing medical students from accessing mental health services, addressing a critical gap in understanding the interplay between mental health prevalence and service utilization barriers in a Saudi Arabian context.

## 2. Methodology

### 2.1. Study design

This was a cross-sectional study conducted among medical students of Jazan University, Saudi Arabia, from September 2023 to March 2024.

### 2.2. Study setting

The study was carried out at faculty of medicine, Jazan University. Jazan University is 1 of the Saudi Arabia kingdom’s 34 universities, which was founded in 1426. According to the annual report issued by Jazan University, the total university population is around 45,000 students, of which 1062 attend the College of Medicine.

### 2.3. Sample size and sampling technique

The minimum required sample size was calculated using the Raosoft sample size calculator (http://www.raosoft.com/samplesize.html), assuming a total population of 1062 students, a 95% confidence interval, a 4% margin of error, and a 50% response distribution. This calculation yielded an initial sample size of 384. To account for a 10% nonresponse rate, the sample size was adjusted to 427 participants. The study successfully recruited 390 participants, achieving a response rate of 91.3%, which exceeds the base requirement and ensures adequate statistical power for the analysis. A convenience sampling technique was used to recruit participants.

### 2.4. Inclusion and exclusion criteria

The study included medical students from the 2nd to 6th year at Jazan University. Students in the 1st year (who had not yet commenced basic medical sciences), nonmedical students, and those who refused to participate were excluded from the study.

### 2.5. Data collection process and tool

Data were collected using a structured, self-administered online questionnaire designed to assess symptoms of depression, anxiety, and stress, as well as barriers to seeking mental health care among medical students. The questionnaire included the Arabic version of the validated depression, anxiety, and stress scale (DASS-21), which has demonstrated high internal consistency, with Cronbach alpha coefficients reported as 0.88 for depression, 0.82 for anxiety, and 0.90 for stress in previous validation studies.^[11]^

Additional items related to sociodemographic characteristics and barriers to mental health care were carefully designed based on a review of the existing literature and study objectives.

Before full-scale deployment, the questionnaire was subjected to pretesting to assess its clarity, relevance, and usability. A small group of 10 medical students (excluded from the final analysis) participated in the pretesting phase. Feedback from this process was used to refine the wording and structure of the questions to ensure comprehension and ease of completion. Subsequently, a pilot test was conducted with a sample of 30 medical students (also excluded from the final analysis) to evaluate the feasibility of the questionnaire and the data collection process. The pilot test confirmed the reliability of the questionnaire and identified minor adjustments needed to improve response rates and reduce ambiguity.

The final version of the questionnaire was disseminated via social media platforms (e.g., WhatsApp, Telegram, X) and email, ensuring accessibility and convenience for participants. Participation was voluntary, and informed consent was obtained from all participants before data collection. To maintain confidentiality, no personal identifiers, such as names or ID numbers, were collected, and data were stored anonymously in Microsoft Excel.

The DASS-21 includes 21 items, divided into 3 subscales (7 items each) for depression, anxiety, and stress. Participants were required to indicate the presence of symptoms over the previous week on a scale from 0 (did not apply to me at all) to 3 (applied to me very much, or most of the time). Scores on each subscale were multiplied by 2 for interpretation, as the DASS-21 is a shortened version of the DASS-42.

Recommended cutoffs for each category were used for interpretation:

- Depression: normal (0–9), mild (10–13), moderate (14–20), severe (21–27), extremely severe (≥28).
- Anxiety: normal (0–7), mild (8–9), moderate (10–14), severe (15–19), extremely severe (≥20).
- Stress: normal (0–14), mild (15–18), moderate (19–25), severe (26–33), extremely severe (≥34).

These scores were compared across participants with different sociodemographic backgrounds to explore associations and variations.

### 2.6. Statistical analysis

The collected data were analyzed using International Business Machines Corporation Statistical Package for the Social Sciences version 27.0.1 (Chicago). Descriptive statistics were employed to summarize the sociodemographic characteristics and mental health outcomes of the participants. For categorical variables, frequencies and percentages were calculated, while continuous variables were summarized using either means with standard deviations or medians with interquartile ranges (IQRs), depending on the distribution of the data. The normality of the data was assessed using the Kolmogorov–Smirnov test, which indicated a non-normal distribution (*P* < .001). As a result, medians and IQRs were primarily reported for continuous variables.

To explore differences in depression, anxiety, and stress scores across sociodemographic groups, nonparametric tests were applied due to the non-normal distribution of the data. The Mann–Whitney *U* test was used to compare scores between 2 independent groups, while the Kruskal–Wallis test was applied for comparisons involving more than 2 groups. A *P*-value of < .05 was considered statistically significant, indicating a 95% confidence interval for all analyses.

### 2.7. Institutional review board statement

Ethical approval was obtained from the Standing Committee for Scientific Research – Jazan University (HAPO-10-Z-001) (reference no.: REC-45/05/861 on December 04, 2023). Furthermore, permission for data collection was secured from pertinent faculty of medicine. This study adhered to ethical standards within the geographical boundaries of the Kingdom of Saudi Arabia. Participants’ anonymity was prioritized, and strict confidentiality was maintained for all collected questionnaires.

## 3. Results

### 3.1. Sociodemographic characteristics and estimated scores for depression, anxiety and stress among the study participants

The study encompassed 390 participants, with a nearly equal distribution across gender, comprising 50.5% males and 49.5% females (see Table 1). The majority of participants fell within the age range of 21 to 23 years (62.1%), followed by those aged 18 to 20 years (23.6%), with a smaller proportion aged 24 or older (14.4%). Concerning residency, a larger percentage hailed from villages (60.8%) compared to urban areas (39.2%). Most participants reported being single (94.9%), while a minority were either married (4.6%) or divorced (0.5%). Regarding academic status, the highest representation was observed among 4th-year students (24.6%), followed by 5th-year students (21.0%), with relatively similar proportions across other academic years (2nd year: 14.6%, 3rd year: 19.7%, 6th year: 20.0%). Median scores for depression, anxiety, and stress were 14.00, 12.00, and 16.00, respectively, with interquartile ranges (IQR) of 4.00 to 22.00, 4.00 to 20.00, and 6.00 to 24.00, indicating substantial variability in symptom severity.

**Table 1:** Sociodemographic characteristics and estimated scores for depression, anxiety and stress among the study participants.

		N	%
Gender	Male	197	50.5%
	Female	193	49.5%
Age	18 to 20	92	23.6%
	21 to 23	242	62.1%
	≥24	56	14.4%
Place of residence	Village	237	60.8%
	City	153	39.2%
Marital status	Single	370	94.9%
	Married	18	4.6%
	Divorced	2	0.5%
Year of Study in Medical School	2nd Year	57	14.6%
	3rd Year	77	19.7%
	4th Year	96	24.6%
	5th Year	82	21.0%
	6th Year	78	20.0%
Estimated score	Depression	14.00^M*^	4.00 to 22.00^I*^
	Anxiety	12.00^M*^	4.00 to 20.00^I*^
	Stress	16.00^M*^	6.00 to 24.00^I*^

Categorical variables were expressed in number (N) and percentage (%) and the continuous variables were expressed in median (M*) and Interquartile Ranges (I*).

### 3.2. Prevalence of depression, anxiety and stress among the study participants

**Figure 1.**
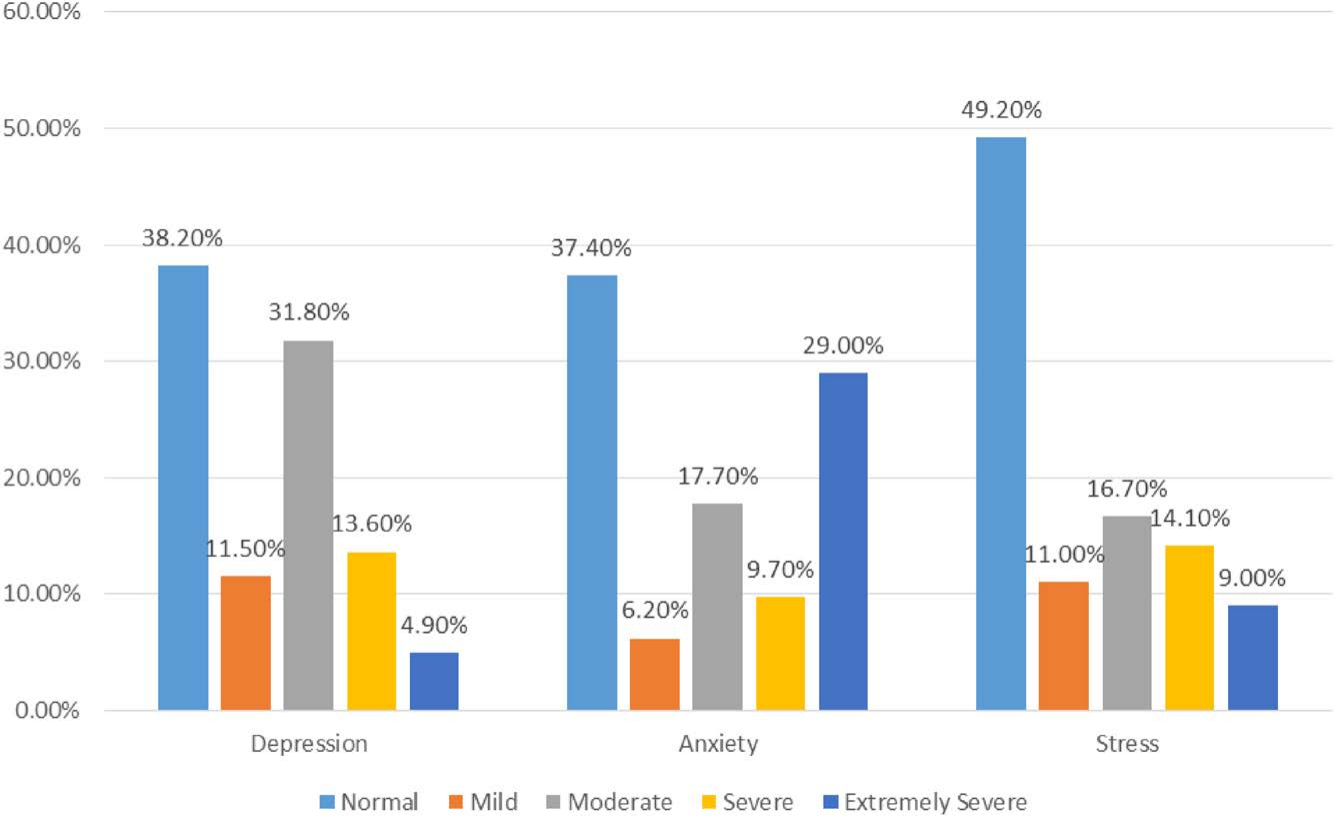
Prevalence of depression, anxiety and stress among the participants.

The study findings revealed a considerable prevalence of depression, anxiety, and stress symptoms among the participants (Fig. 1). Analysis of depression categories showed that 38.2% of participants fell within the normal range, while notable proportions experienced mild (11.5%), moderate (31.8%), severe (13.6%), or extremely severe (4.9%) depression. Similarly, for anxiety, 37.4% were categorized as normal, while substantial percentages exhibited symptoms ranging from mild (6.2%) to extremely severe (29.0%). Regarding stress, 49.2% were classified as normal, whereas significant portions reported mild (11.0%), moderate (16.7%), severe (14.1%), or extremely severe (9.0%) stress levels.

### 3.3. Participants’ response and reflections to the DASS scale items

The students were answered the 21 questions of the DASS Scale, indicating the presence of symptoms over the previous week. Among the symptoms of depression; 14.1% of the participants felt sad and distressed most of the time and 15.9% of them experienced loss of energy when doing things. Regarding symptoms of anxiety; 13.1% of the participants afraid of situations in which they might lose control or feel embarrass and 10.8% felt trembling most of the time. When come to the stress category; 15.1% of the participants found it difficult to take initiative in doing things most of the time and 13.1% tend to overreact to circumstances and events. Other participants’ response and reflections were presented in Appendix 1, Supplemental Digital Content, http://links.lww.com/MD/O254.

### 3.4. Barriers against seeking mental health services

**Figure 2.**
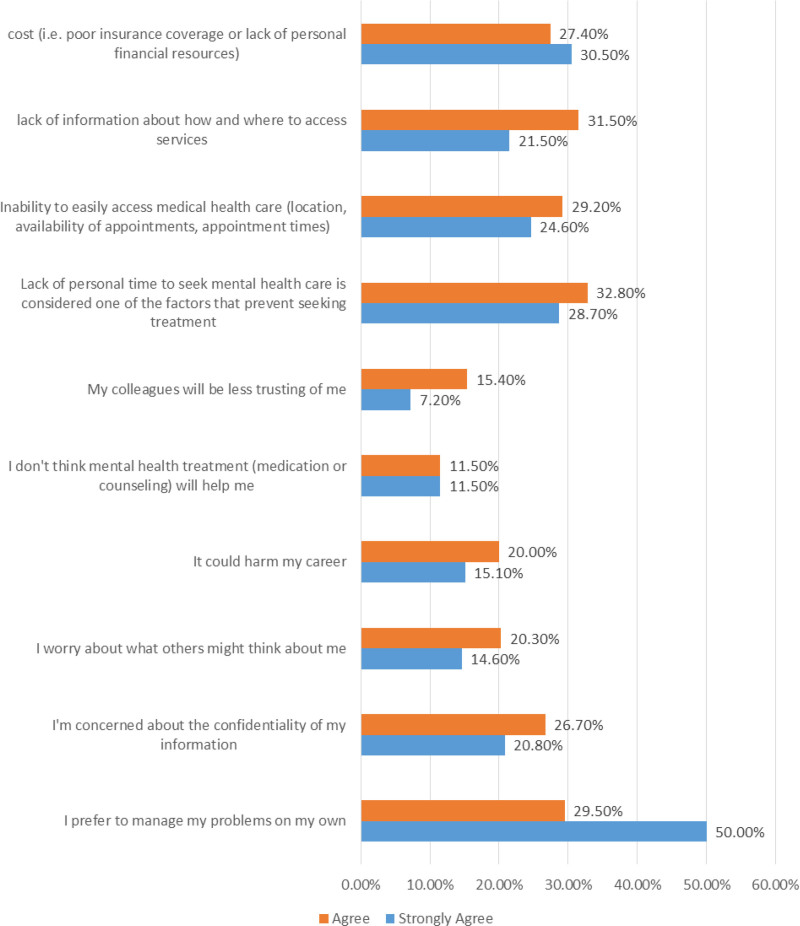
Barriers against seeking mental health services among the participants.

The participants identified various barriers to seeking mental health treatment, with notable proportions expressing agreement or strong agreement with specific concerns (Table 2 and Fig. 2). The most prevalent barrier was the preference to manage problems independently, with 79.5% of participants either agreeing or strongly agreeing. Concerns about confidentiality ranked second, with 47.5% expressing agreement or strong agreement, followed closely by worries about what others might think (34.9%). Additionally, apprehensions about potential harm to one’s career were cited by 35.1% of participants. A considerable portion also expressed skepticism regarding the effectiveness of mental health treatment, with 23.0% either agreeing or strongly agreeing. Concerns about colleagues’ trust levels were voiced by 22.6% of participants. Moreover, logistical challenges such as lack of personal time (61.5%), difficulty in accessing services (53.8%), and insufficient information about service availability (53.0%) were reported. Cost-related barriers were also significant, with 57.9% of participants expressing agreement or strong agreement with this concern.

**Table 2:** Barriers to seeking mental health treatment according to the participants.

	Strongly disagree		Disagree		Neutral		Agree		Strongly agree	
	N	%	N	%	N	%	N	%	N	%
I prefer to manage my problems on my own	7	1.8%	19	4.9%	54	13.8%	115	29.5%	195	50.0%
I’m concerned about the confidentiality of my information	36	9.2%	77	19.7%	92	23.6%	104	26.7%	81	20.8%
I worry about what others might think about me	60	15.4%	76	19.5%	118	30.3%	79	20.3%	57	14.6%
It could harm my career	60	15.4%	90	23.1%	103	26.4%	78	20.0%	59	15.1%
I don’t think mental health treatment (medication or counseling) will help me	110	28.2%	99	25.4%	91	23.3%	45	11.5%	45	11.5%
My colleagues will be less trusting of me	104	26.7%	105	26.9%	93	23.8%	60	15.4%	28	7.2%
Lack of personal time to seek mental health care is considered 1 of the factors that prevent seeking treatment	31	7.9%	38	9.7%	81	20.8%	128	32.8%	112	28.7%
Inability to easily access medical health care (location, availability of appointments, appointment times)	40	10.3%	56	14.4%	84	21.5%	114	29.2%	96	24.6%
Lack of information about how and where to access services	46	11.8%	53	13.6%	84	21.5%	123	31.5%	84	21.5%
cost (i.e. poor insurance coverage or lack of personal financial resources)	44	11.3%	38	9.7%	82	21.0%	107	27.4%	119	30.5%

### 3.5. The association between depression scores and sociodemographic factors of the participants

Table 3 provides an analysis of the association between depression scores and various sociodemographic factors among participants. There is a statistically significant difference in depression scores between females (Mean depression score = 15.78) and males (mean depression score = 13.01), with females showing higher mean scores than males (*P*-value of .031). There is no statistically significant difference in depression scores among different age groups (*P*-value = .346), the year of study in medical school (*P*-value = .213) and the place of residence (city vs village) does not significantly impact depression scores (*P*-value = .269). Regarding marital status, there is no significant difference in depression scores (*P*-value = .389), although married individuals have the highest mean scores (17.22), and divorced individuals have the lowest (7.00).

**Table 3:** Association of depression with sociodemographic characteristics.

		Depression score				
		Mean	SD	Median	IQR	*P*-value^U/K^
Gender	Female	15.78	12.17	14.00	4.00 to 24.00	.031*
	Male	13.01	10.78	12.00	4.00 to 20.00	
Age	≥24	16.93	13.45	16.00	6.00 to 25.00	.346
	18 to 20	14.76	11.96	14.00	4.00 to 23.00	
	21 to 23	13.64	10.88	12.00	4.00 to 22.00	
Place of residence	City	13.44	10.94	12.00	4.00 to 20.00	.269
	Village	14.99	11.92	14.00	4.00 to 24.00	
Marital status	Divorced	7.00	9.90	7.00	0.00 to 14.00	.389
	Married	17.22	12.71	13.00	8.00 to 24.00	
	Single	14.28	11.51	14.00	4.00 to 22.00	
Year of Study in Medical School	2nd Year	14.18	11.85	12.00	4.00 to 24.00	.213
	3rd Year	15.09	12.58	14.00	2.00 to 22.00	
	4th Year	11.94	9.50	12.00	4.00 to 18.00	
	5th Year	16.24	12.04	15.00	4.00 to 26.00	
	6th Year	14.87	11.87	14.00	4.00 to 24.00	

U Mann–Whitney *U* test, ^K^Kruskal–Wallis test.

IQR = interquartile range, SD = standard deviation.

* *P* < .05, significant.

### 3.6. The association between Anxiety scores and sociodemographic factors of the participants

Table 4 presents the association between anxiety scores and various sociodemographic factors among participants. There is a statistically significant difference (*P*-value = <.001) in anxiety scores between females and males, with the females (mean anxiety score = 15.21) have higher anxiety scores compared to males (mean anxiety score = 10.73). There is no significant difference in anxiety scores based on different age groups (*P*-value = .171), place of residence (*P*-value = .098) and marital status (*P*-value = .170), although participants from age group ≥ 24, those from villages and married individuals tend to have the highest mean anxiety scores.

**Table 4:** Association of anxiety with sociodemographic characteristics.

				Anxiety score			
		Mean	SD		Median	IQR	*P*-value^U/K^
Gender	Female	15.21	11.16		14.00	4.00 to 24.00	<.001*
	Male	10.73	9.80		8.00	2.00 to 18.00	
Age	≥24	15.18	12.10		13.00	4.00 to 22.00	.171
	18 to 20	13.74	10.54		14.00	4.00 to 20.00	
	21 to 23	12.13	10.39		10.00	2.00 to 20.00	
Place of residence	City	11.91	10.57		10.00	2.00 to 18.00	.098
	Village	13.62	10.78		12.00	4.00 to 22.00	
Marital status	Divorced	3.00	4.24		3.00	0.00 to 6.00	.170
	Married	16.67	12.65		15.00	6.00 to 30.00	
	Single	12.82	10.60		12.00	4.00 to 20.00	
Year of Study in Medical School	2nd Year	13.89	10.31		14.00	6.00 to 20.00	.273
	3rd Year	13.95	11.31		14.00	4.00 to 22.00	
	4th Year	10.52	9.01		8.00	3.00 to 16.00	
	5th Year	13.66	10.88		12.00	4.00 to 20.00	
	6th Year	13.51	11.93		12.00	2.00 to 22.00	

U Mann–Whitney *U* test, ^K^Kruskal–Wallis test.

IQR = interquartile range, SD = standard deviation.

* *P* < .05, significant.

*The association between Anxiety scores and sociodemographic factors of the participants:* Table 5 presents the relationship between stress scores and various sociodemographic factors among participants. As same as in depression and anxiety, females having higher stress scores than males with (*P*-value = .030) indicating a statistically significant difference in stress scores between females (mean stress score = 17.72) and males (mean stress score = 15.11). Although participants aged ≥ 24 tend to have higher stress scores (mean = 19.7, the difference is not statistically significant (*P*-value = .062). The place of residence (city vs village) does not significantly affect stress scores (*P*-value = .199), although those living in villages have slightly higher scores (mean = 17.01) compared to those living in city (mean = 15.45). While there is no statistically significant difference in stress scores by marital status (*P*-value = .135), married participants tend to have higher stress scores (mean = 18.44) compared to singles (mean = 16.37) and divorced individuals (mean = 3.00). The year of study in medical school does not significantly impact stress scores (*P*-value = .477), although there is a trend of higher stress levels among 2nd (mean = 17.51, IQR = 6.00 to 28.00) and 5th-year (mean = 17.44, IQR = 8.00–26.00) students.

**Table 5:** Association of stress with sociodemographic characteristics.

		Stress score				
		Mean	SD	Median	IQR	*P*-value^U/K^
Gender	Female	17.72	11.28	16.00	8.00 to 26.00	.030*
	Male	15.11	10.81	14.00	6.00 to 22.00	
Age	≥24	19.71	11.14	19.00	14.00 to 27.00	.062
	18 to 20	16.30	11.54	14.00	6.00 to 25.00	
	21 to 23	15.67	10.84	14.00	6.00 to 24.00	
Place of residence	City	15.45	10.93	14.00	6.00 to 22.00	.199
	Village	17.01	11.20	16.00	8.00 to 24.00	
Marital status	Divorced	3.00	4.24	3.00	0.00 to 6.00	.135
	Married	18.44	11.73	23.00	6.00 to 26.00	
	Single	16.37	11.07	16.00	6.00 to 24.00	
Year of Study in Medical School	2nd Year	17.51	11.91	16.00	6.00 to 28.00	.477
	3rd Year	16.60	12.25	16.00	6.00 to 26.00	
	4th Year	14.54	9.90	14.00	6.00 to 20.00	
	5th Year	17.44	11.02	15.00	8.00 to 26.00	
	6th Year	16.59	10.81	18.00	6.00 to 24.00	

U Mann–Whitney *U* test, ^K^Kruskal–Wallis test.

IQR = interquartile range, SD = standard deviation.

* *P* < .05, significant.

These findings suggest a gender-based disparity in the prevalence of depression, anxiety, and stress among the participants, emphasizing the need for targeted interventions and support mechanisms, especially for female students.

## 4. Discussion

Unmet mental health needs have a substantial negative impacts on student health and productivity. Studying mental health among medical university students is vital not only for their personal well-being but also for their future roles as healthcare providers and the overall improvement of healthcare delivery. By fostering a supportive environment and promoting mental health awareness, medical universities can help students thrive academically, professionally, and personally. This study intended to determine the prevalence of symptoms of depression, anxiety, and stress among the medical students and to identify the barriers preventing them from seeking help when needed.

## 5. Prevalence of mental health symptoms

The findings revealed considerable variability in symptom severity, with a substantial proportion of students experiencing moderate to extremely severe symptoms. Gender was the only sociodemographic factor consistently associated with mental health outcomes, with females reporting higher levels of depression, anxiety, and stress compared to males. These findings align with previous studies in Saudi Arabia and globally.^[12,13]^

A study by Kulsoom and Afsar (2015), conducted in a multiethnic setting in Saudi Arabia, reported high levels of stress, anxiety, and depression among medical students, with female students showing higher levels of distress compared to males.^[14]^ Similarly, a study by Inam (2007) found that anxiety and depression were prevalent among Saudi medical students, with significant gender differences in mental health outcomes.^[15]^ Research conducted by Ibrahim et al (2013) among medical students in Saudi Arabia found similar patterns of mental health issues, emphasizing the need for targeted interventions.^[16]^ The present study’s findings add to this body of evidence, emphasizing the persistent and widespread nature of mental health challenges among female medical students.

Further comparison with the study by Sultan et al (2022), which investigated mental health among students at Umm-Al-Qura University during the COVID-19 pandemic, highlights the unique challenges faced by medical students. Sultan et al reported a high prevalence of depression (45%), anxiety (39.4%), and stress (36.4%) among medical and laboratory students, closely mirroring the findings of this study.^[17]^ While the present study was conducted in a non-pandemic context, the comparably high rates of psychological distress underscore the need for targeted interventions to address systemic issues contributing to poor mental health.

Globally, a meta-analysis by Rotenstein et al (2016) highlighted the high prevalence of depression and anxiety among medical students globally, underscoring the importance of addressing mental health concerns in this population.^[18]^ A recent study among university students in Bangladesh revealed that 71 % of university students experience a moderate to severe level of anxiety, 43 % encounter a moderate to severe level of depression, and 25.61 % grapple with a moderate to severe level of stress.^[19]^

## 6. Barriers to mental health treatment

This study identified several barriers to seeking mental health care, including a preference for self-management, concerns about confidentiality, societal stigma, and logistical challenges. This finding is consistent with previous research demonstrating a tendency among medical students to self-manage mental health issues.^[14,20–22]^ For instance, Kulsoom and Afsar (2015) noted that cultural norms and stigma often prevent students from seeking help, despite experiencing significant mental health challenges.

Confidentiality concerns were particularly prominent in this study, reinforcing findings from Sultan et al (2022), who emphasized the importance of anonymous and nonjudgmental mental health services to facilitate help-seeking behavior among students.^[17]^ This aligns with the findings of studies by Eigenhuis et al (2021) and Varma et al (2022), which emphasize the importance of confidentiality in facilitating help-seeking behavior among medical students.^[23,24]^

Worries about what others might think and apprehensions regarding potential harm to one’s career were also significant barriers, reflecting the stigma surrounding mental health issues in academic and professional settings. Addressing stigma through awareness campaigns and educational initiatives is essential in creating a supportive environment conducive to seeking mental health treatment.^[25,26]^

Skepticism regarding the effectiveness of mental health treatment and concerns about colleagues’ trust levels further contribute to the complexity of help-seeking behavior among medical students. Providing evidence-based information about the efficacy of mental health interventions and fostering a culture of support and trust within the medical community can help mitigate these barriers.^[27]^

Logistical challenges, including lack of personal time, difficulty in accessing services, and insufficient information about service availability, highlight systemic barriers that need to be addressed at the institutional level. Moreover, the significant cost-related barriers underscore the importance of ensuring affordable and accessible mental health services for medical students.^[28]^

## 7. Associations with sociodemographic factors

Regarding the associations between depression, anxiety, and stress scores and various sociodemographic characteristics, findings revealed significant gender-based differences in the prevalence of depression, anxiety, and stress, with females exhibiting higher mean scores compared to males. These results are consistent with previous research highlighting gender disparities in mental health outcomes among university students.^[29–31]^ The study underscores the importance of recognizing and addressing gender-specific factors contributing to mental health disparities among medical students.

Age, marital status, place of residence and year of study in medical school do not show significant associations with depression, anxiety, and stress scores in this study. While there are trends indicating higher stress in older, married students and slightly elevated scores in specific years of study, these differences are not statistically significant. Ayoob et al, in the study conducted in college of medicine, King Faisal University, Saudi Arabia reported that; quality of life of medical students may influenced by gender, academic year, marital status, monthly income and family type.^[32]^ While these findings suggest that gender may be a primary determinant of mental health outcomes among medical students, further research could explore these trends in larger or more diverse samples to determine if these patterns hold or if other variables might interact with the gender to impact on mental health outcomes.

## 8. Implications and future directions

Study has several implications for practice and directions for future research. Recognizing the gender-based disparities in mental health outcomes, particularly the higher prevalence of depression, anxiety, and stress among female students, calls for the implementation of gender-sensitive mental health interventions. These interventions should address the unique needs and concerns of female medical students, aiming to reduce stigma, enhance accessibility, and promote help-seeking behavior. Addressing the barriers identified, such as concerns about confidentiality, logistical challenges, and skepticism regarding the effectiveness of mental health treatment, is crucial in improving access to mental health services. Future research endeavors could focus on longitudinal studies to track changes in mental health outcomes over time, qualitative investigations to explore students’ experiences and perceptions, and comparative studies across different universities or countries to identify institutional or cultural factors influencing mental health. Moreover, intervention studies evaluating the effectiveness of various mental health interventions and examining the role of faculty and institutional support in promoting well-being among medical students are warranted.

## 9. Limitations

This study has several limitations that warrant consideration. The cross-sectional design limits the ability to establish causal relationships between sociodemographic factors and mental health outcomes among medical students. While this approach provides a snapshot of the prevalence of depression, anxiety, and stress, longitudinal studies are needed to explore the dynamic and evolving nature of mental health within this population.

The study employed a convenience sampling technique, which, although practical for recruitment, may introduce selection bias and limit the generalizability of the findings. A probability-based sampling approach, such as simple random or systematic random sampling, would have ensured a more representative sample of the target population and enhanced the validity of the results.

Additionally, the study relied on the DASS-21 screening tool to assess symptoms of depression, anxiety, and stress. While the DASS-21 is a validated and widely used instrument, it serves as a screening tool rather than a diagnostic tool. As such, students categorized as having depression, anxiety, or stress based on their DASS-21 scores would require further psychiatric evaluation using comprehensive clinical inventories and diagnostic methods to confirm these conditions. This limitation highlights the importance of integrating clinical assessments into future research to validate the findings.

Moreover, the use of self-reported data introduces the potential for response bias, as participants may underreport or over report their symptoms due to social desirability or personal perception. Incorporating objective assessments, such as clinical interviews, in future studies could mitigate this limitation and provide a more nuanced understanding of mental health among medical students.

Finally, the study was conducted at a single University, limiting the generalizability of the results to other institutions and regions. Expanding the scope of future research to include multiple universities or diverse populations would offer a more comprehensive perspective on the mental health challenges faced by medical students.

By addressing these limitations in future research, more robust and generalizable insights can be gained, ultimately contributing to improved mental health support and interventions for medical students.

## 10. Conclusions

This study highlights the significant prevalence of depression, anxiety, and stress symptoms among medical students at Jazan University. Median scores for depression, anxiety, and stress were 14.00, 12.00, and 16.00, respectively, indicating notable levels of psychological distress within this population. A considerable proportion of students experienced moderate to severe symptoms across all 3 domains, with 31.8% categorized as having moderate depression, 29.0% with extremely severe anxiety, and 16.7% with moderate stress. Female students exhibited higher levels of psychological distress compared to their male counterparts, emphasizing the need for gender-sensitive mental health interventions. Additionally, barriers to accessing mental health care, including a preference for self-management, concerns about confidentiality, societal judgment, and fears of career-related repercussions, were identified. These findings underscore the necessity for tailored interventions to reduce stigma and enhance the accessibility of mental health services for medical students.

## Acknowledgments

The authors are grateful to the students for their generous support and participation in this study. The authors are gratefully acknowledge the funding of the Deanship of Graduate Studies and Scientific Research, Jazan University, Saudi Arabia, through Project Number: GSSRD-24.

## Author contributions

**Conceptualization:** Osama Albasheer, Essam Al Ageeli, Turki I. Aljezani, Khalid A. Bakri, Salman M. Jathmi, Abdullah Maashi, Ahmad Abo Khirat, Ali W. Hakami, Arif A. Haddadi, Suhaila Ali, Amani Abdelmola, Anas Ahmed.

**Data curation:** Osama Albasheer, Essam Al Ageeli, Turki I. Aljezani, Khalid A. Bakri, Salman M. Jathmi, Abdullah Maashi, Ahmad Abo Khirat, Ali W. Hakami, Arif A. Haddadi, Suhaila Ali, Amani Abdelmola, Anas Ahmed.

**Formal analysis:** Osama Albasheer, Essam Al Ageeli, Turki I. Aljezani, Khalid A. Bakri, Salman M. Jathmi, Abdullah Maashi, Ahmad Abo Khirat, Ali W. Hakami, Arif A. Haddadi, Suhaila Ali, Amani Abdelmola, Anas Ahmed.

**Funding acquisition:** Osama Albasheer, Essam Al Ageeli, Turki I. Aljezani, Khalid A. Bakri, Salman M. Jathmi, Abdullah Maashi, Ahmad Abo Khirat, Ali W. Hakami, Arif A. Haddadi, Suhaila Ali, Amani Abdelmola, Anas Ahmed.

**Investigation:** Osama Albasheer, Essam Al Ageeli, Turki I. Aljezani, Khalid A. Bakri, Salman M. Jathmi, Abdullah Maashi, Ahmad Abo Khirat, Ali W. Hakami, Arif A. Haddadi, Suhaila Ali, Amani Abdelmola, Anas Ahmed.

**Methodology:** Osama Albasheer, Essam Al Ageeli, Turki I. Aljezani, Khalid A. Bakri, Salman M. Jathmi, Abdullah Maashi, Ahmad Abo Khirat, Ali W. Hakami, Arif A. Haddadi, Suhaila Ali, Amani Abdelmola, Anas Ahmed.

**Project administration:** Osama Albasheer, Essam Al Ageeli, Turki I. Aljezani, Khalid A. Bakri, Salman M. Jathmi, Abdullah Maashi, Ahmad Abo Khirat, Ali W. Hakami, Arif A. Haddadi, Suhaila Ali, Amani Abdelmola, Anas Ahmed.

**Resources:** Osama Albasheer, Essam Al Ageeli, Turki I. Aljezani, Khalid A. Bakri, Salman M. Jathmi, Abdullah Maashi, Ahmad Abo Khirat, Ali W. Hakami, Arif A. Haddadi, Suhaila Ali, Amani Abdelmola, Anas Ahmed.

**Software:** Osama Albasheer, Essam Al Ageeli, Turki I. Aljezani, Khalid A. Bakri, Salman M. Jathmi, Abdullah Maashi, Ahmad Abo Khirat, Ali W. Hakami, Arif A. Haddadi, Suhaila Ali, Amani Abdelmola, Anas Ahmed.

**Supervision:** Osama Albasheer, Essam Al Ageeli, Turki I. Aljezani, Khalid A. Bakri, Salman M. Jathmi, Abdullah Maashi, Ahmad Abo Khirat, Ali W. Hakami, Arif A. Haddadi, Suhaila Ali, Amani Abdelmola, Anas Ahmed.

**Validation:** Osama Albasheer, Essam Al Ageeli, Turki I. Aljezani, Khalid A. Bakri, Salman M. Jathmi, Abdullah Maashi, Ahmad Abo Khirat, Ali W. Hakami, Arif A. Haddadi, Suhaila Ali, Amani Abdelmola, Anas Ahmed.

**Visualization:** Osama Albasheer, Essam Al Ageeli, Turki I. Aljezani, Khalid A. Bakri, Salman M. Jathmi, Abdullah Maashi, Ahmad Abo Khirat, Ali W. Hakami, Arif A. Haddadi, Suhaila Ali, Amani Abdelmola, Anas Ahmed.

**Writing – original draft:** Osama Albasheer, Essam Al Ageeli, Turki I. Aljezani, Khalid A. Bakri, Salman M. Jathmi, Abdullah Maashi, Ahmad Abo Khirat, Ali W. Hakami, Arif A. Haddadi, Suhaila Ali, Amani Abdelmola, Anas Ahmed.

**Writing – review & editing:** Osama Albasheer, Essam Al Ageeli, Turki I. Aljezani, Khalid A. Bakri, Salman M. Jathmi, Abdullah Maashi, Ahmad Abo Khirat, Ali W. Hakami, Arif A. Haddadi, Suhaila Ali, Amani Abdelmola, Anas Ahmed.

## Supplementary Material


