# Supplementary material for: Prevalence of depressive, anxiety, and stress symptoms and barriers to mental health services among medical students at Jazan University, Saudi Arabia: A cross-sectional study
Source: Medicine (Baltimore). 2025 Jan 3;104(1):e41185. doi: 10.1097/MD.0000000000041185 (PMC11709213; doi:10.1097/MD.0000000000041185)
Supplement: Supplementary file 1 [file medi-104-e41185-s001.docx]

**Appendix 1:** Responses of the participants to individual items of the DASS (Depression, Anxiety, and Stress) scale

|  | Did not apply to me at all | | Applied to me to some degree, or some of the time | | Applied to me to a considerable degree or a good part of time | | Applied to me very much or most of the time | |
| --- | --- | --- | --- | --- | --- | --- | --- | --- |
|  | **N** | **%** | **N** | **%** | **N** | **%** | **N** | **%** |
| 1. I found it difficult to relax and unwind | 94 | 24.1% | 164 | 42.1% | 94 | 24.1% | 38 | 9.7% |
| 2. My throat felt dry | 158 | 40.5% | 132 | 33.8% | 65 | 16.7% | 35 | 9.0% |
| 3. I didn't seem to be able to feel positive emotions at all | 146 | 37.4% | 142 | 36.4% | 68 | 17.4% | 34 | 8.7% |
| 4. I felt difficulty breathing (extreme rapid breathing, panting without physical effort, for example) | 194 | 49.7% | 103 | 26.4% | 61 | 15.6% | 32 | 8.2% |
| 5. I found it difficult to take initiative in doing things | 107 | 27.4% | 123 | 31.5% | 101 | 25.9% | 59 | 15.1% |
| 6. I tend to overreact to circumstances and events | 139 | 35.6% | 109 | 27.9% | 91 | 23.3% | 51 | 13.1% |
| 7. I felt a trembling (in my hands, for example) | 179 | 45.9% | 96 | 24.6% | 73 | 18.7% | 42 | 10.8% |
| 8. Depression, Anxiety, and Stress Scale (DASS-21) Use the phone in landscape mode [I felt like I was consuming a lot of nervous energy (nervous energy is a surge of energy you feel when you are anxious or stressed) | 102 | 26.2% | 118 | 30.3% | 92 | 23.6% | 78 | 20.0% |
| 9. I was afraid of situations in which I might lose control of my temper and embarrass myself | 163 | 41.8% | 108 | 27.7% | 68 | 17.4% | 51 | 13.1% |
| 10. I felt like I had nothing to look forward to | 171 | 43.8% | 109 | 27.9% | 60 | 15.4% | 50 | 12.8% |
| 11. I felt disturbed and upset | 117 | 30.0% | 132 | 33.8% | 87 | 22.3% | 54 | 13.8% |
| 12. Depression, Anxiety, and Stress Scale (DASS-21) Use the phone in landscape mode [I find it difficult to relax | 120 | 30.8% | 136 | 34.9% | 93 | 23.8% | 41 | 10.5% |
| 13. I felt sad and distressed | 115 | 29.5% | 127 | 32.6% | 93 | 23.8% | 55 | 14.1% |
| 14. I was intolerant of anything that kept me from continuing what I was doing | 158 | 40.5% | 119 | 30.5% | 77 | 19.7% | 36 | 9.2% |
| 15. I felt like I was about to fall into a state of sudden terror (for no reason) | 205 | 52.6% | 97 | 24.9% | 59 | 15.1% | 29 | 7.4% |
| 16. I lost the feeling of enthusiasm for anything | 146 | 37.4% | 122 | 31.3% | 69 | 17.7% | 53 | 13.6% |
| 17. I felt like I had little value as a person | 204 | 52.3% | 92 | 23.6% | 58 | 14.9% | 36 | 9.2% |
| 18. I felt like I tended to get angry quickly (rage is a feeling of extreme anger over an insult someone inflicts on you) | 134 | 34.4% | 116 | 29.7% | 78 | 20.0% | 62 | 15.9% |
| 19. I felt my heart beating without physical effort (increased heart rate, or absence of a heartbeat, for example) | 159 | 40.8% | 97 | 24.9% | 75 | 19.2% | 59 | 15.1% |
| 20. I felt afraid for no good reason | 187 | 47.9% | 101 | 25.9% | 61 | 15.6% | 41 | 10.5% |
| 21. I felt like life had no meaning | 203 | 52.1% | 88 | 22.6% | 55 | 14.1% | 44 | 11.3% |
